# What Explains the Association between Usage of Social Networking Sites (SNS) and Depression Symptoms? The Mediating Roles of Self-Esteem and Fear of Missing Out

**DOI:** 10.3390/ijerph18083916

**Published:** 2021-04-08

**Authors:** Angel Nga Man Leung, Wilbert Law, Yvonne Yiqing Liang, Antony Chun Lam Au, Cheng Li, Henry Kin Shing Ng

**Affiliations:** 1 Department of Psychology and Centre for Psychosocial Health, The Education University of Hong Kong, Hong Kong, China; nmleung@eduhk.hk; 2 Department of Psychology, The Education University of Hong Kong, Hong Kong, China; yvonneleunggg@gmail.com (Y.Y.L.); antony.au.55@gmail.com (A.C.L.A.); s1114362@s.eduhk.hk (C.L.); 3 Department of Psychology, The University of Hong Kong, Hong Kong, China; nghks@hku.hk

**Keywords:** social media, fear of missing out, self-esteem, depression

## Abstract

The goal of the study was to understand the mechanisms of how social networking sites (SNS) usage is related to depression symptoms, as measured by the Center for Epidemiological Studies-Depression Scale (CESD). Three studies were conducted to examine the mediation roles of self-esteem and Fear of Missing Out (FoMO). In Study 1, among 347 Chinese college students, time spent on SNS was negatively associated with self-esteem; while self-esteem then negatively associated with depression symptoms. In Study 2, among 180 Chinese college students, time spent on SNS was positively related to FoMO; while FoMO then positively related to depression symptoms. In Study 3, among 233 Chinese university students, both self-esteem and FoMO were simultaneously included in the mediation model to test their respective roles in explaining depression symptoms. Results showed that more time spent on SNS was related to lower self-esteem, and higher FoMO, respectively; while self-esteem then negatively, and FoMO then positively, explained depression symptoms, respectively. In addition, when participants spent 3.5 h (Study 1), 2.5 h (Study 2), and 2.54 h (Study 3) on SNS, they reached the cutoff for subthreshold depression, as measured by CESD. Combining results from three studies, both self-esteem and FoMO mediated the relation between SNS usage and depression symptoms. This study provides implications to understand the mechanism of SNS-related depression.

## 1. Introduction

Social networking sites (SNS) allow people to communicate, however, research has remained inconclusive as to whether SNS promotes or harms subjective well-being, and its underlying mechanism. While a meta-analysis suggested that the majority of studies on online social technologies resulted in either mixed or no effect(s) on adolescent’s well-being [1], another meta-analysis with 68,964 participants suggested that students who overused the internet tended to have a lower subjective well-being [2]. A couple of mediation research suggested several factors in explaining the relations between SNS usage and psychological well-being [3,4]. Nevertheless, another recent systematic review which studied social media addiction and well-being suggested that the term well-being should be differentiated into positive vs. negative well-being [5]. Indeed, results of this systematic review suggested that negative well-being correlated with addiction scales highly, while other well-being scales had a smaller or no correlation with social media addiction. Therefore, it is worth investigating the relation between SNS usage and negative well-being such as depression symptoms. As “SNS depression” has attracted research attention, this paper focused on how usage of SNS, notably time spent on it, would be related to depression symptoms via fear of missing out (FoMO) and self-esteem (SE). A number of studies suggested the association between social media or SNS usage with depression is a worldwide phenomenon among American, Turkish and Chinese youth [6,7,8]. In a meta-analysis, a small, positive, significant association between using SNS and symptoms of depression was found [9]. Studying the mechanisms of how SNS usage explains depression via mediators will shed light in understanding this relationship. In this paper, we proposed two mediators, self-esteem and FoMO. Three studies were conducted to test the two mediation mechanisms.

### 1.1. SNS, SE, FoMO and Depression

SE is closely related to SNS usage and psychological well-being. SE refers to how individuals evaluate their own worth as a person [10]. SNS offers individuals a platform to communicate, which may modify or maintain their SE as regulated by both reflected appraisal, how individuals perceive others think about them, and social comparison, either upward or downward. Past studies suggested that more usage on Facebook or SNS was associated with lower SE [11]; more depression and anxiety [12]. In addition, frequent Facebook users also reported poorer trait SE, and this was mediated by exposure to upward social comparisons on SNS [13].

FoMO can be viewed as a kind of persistent social anxiety. Przybylski et al. [14] defined FoMO as “*a pervasive apprehension that others might be having rewarding experiences from which one is absent… the desire to stay continually connected with what others are doing*” ([14], p. 1841). As people update their SNS from time to time, it may create a feeling of “missing something important” if one is not checking such updates in time. FoMO was found to be negatively correlated with subjective well-being, positively with depression, and positively with SNS use [15,16,17].

In short, SNS usage has been found to be associated with higher levels of depression and FoMO, and lower self-esteem. Therefore, we hypothesized that there was a direct relationship between SNS usage and depression, with FoMO and self-esteem being possible mediators. This speculation is supported by a study which found FoMO mediating between SNS usage and psychological well-being [18]. As for the mediating role of self-esteem, this speculation is supported by the vulnerability model, which depicts that individuals low in self-esteem are more likely to have depression [19].

### 1.2. Overview of the Three Studies

Three studies involving three independent Chinese populations from Hong Kong, China, were conducted. A three-study design was used here, because when we started conceptualizing studying the mechanism between time spent on SNS and depression, we first would like to test if SE would be a mediator, as past research suggested that SNS usage was associated with lower SE [11] and more depression and anxiety [12] in Study 1. Extending on this thought of looking for a mediator between SNS usage and depression, we further tested if FoMO would be another mediator in Study 2, as past studies also suggested FoMO was positively correlated with SNS usage and more depression [15,16,17]. Finally, we tested if both SE and FoMO mediated SNS usage and depression in Study 3. With three groups of participants being drawn across different timepoints, the generalizability of the results will be increased. As most studies examining the relationship between SNS, SE, FoMO and negative psychological well-being were conducted in the Western context, relatively little is known about the Chinese population. A recent study found heavy social media use among Hong Kong Chinese participants, at or over 2 h per day, was positively associated with probable depression [20]. In Hong Kong, China, those aged 15 to 24 spent the most amount of time on SNS, at an average of 17.7 h per week [21]. As Hong Kong, China, had been under social unrest since June 2019, and communication on social media related to protests is believed to be one of the many factors that may have escalated the social unrest [22]. When young people spend time on SNS, it may be negatively related to their mental health. Therefore, it is particularly timely and meaningful to understand the relationship between negative psychological well-being (i.e., depression) and SNS usage, among Hong Kong Chinese students.

Age and gender were included in each of the studies as covariates because gender differences were found in self-esteem in a meta-analysis [23], but no gender differences were found on FoMO [24]. Nevertheless, age effect was found on FoMO [24]. Therefore, we would like to control for the effects of age and gender in our three hypotheses:

The hypotheses of the three studies were:

Study 1: Hypothesis 1-SNS usage, operationalized as time spent on SNS, would explain higher depression symptoms through its negative effect on SE.
Study 2: Hypothesis 2-SNS usage would explain higher depression symptoms through its positive effect on FoMO.
Study 3: Hypothesis 3-SNS usage would explain higher depression symptoms through both SE and FoMO.

## 2. Study 1

### 2.1. Methods

#### 2.1.1. Participants

A total of 347 Hong Kong Chinese undergraduate students aged between 18 and 28 (35.2% males, 64.8% females) with a mean age of 20.27 (*SD* = 2.02) years joined the study.

#### 2.1.2. Procedures

Ethics approval was obtained from the Research Ethics Board from the university of the first author. Participants were recruited via mass emails to all students via intranet, with no limitations on years of study nor major being set. Students who were interested to join would register, read the information sheet, and consented to complete a questionnaire, which included the measures described below. They received HK$50 (~USD7) for completing the questionnaire. All reliabilities or Cronbach’s alphas shown below are from the current studies.

##### Basic Demographics

Basic demographics including age and gender (Male = 0; Female = 1) were measured.

##### Time Spent on SNS

Participants were asked to report the amount of time they spent on SNS, including applications like Facebook and Instagram per day on a 30-min interval scale. The scale ranged from 0 min to 6.5 h or above. For participants who rated 6.5 h or above of usage, their usage was treated as 6.5 h in the analysis.

##### Self Esteem

Participants self-rated the 10-item Rosenberg SE Scale on a 4-point Likert scale [25]. It assessed the general SE of individuals. A composite score of the scale was used, with a higher score indicating higher degree of SE. A sample item is “On the whole, I am satisfied with myself”. The reliability of this scale for the current study was: α = 0.84.

##### Depression

Depressive symptoms were assessed by the 20-item Center for Epidemiologic Studies Depression Scale (CES-D) [26]. Participants self-rated whether they had symptoms of depression, on a scale from 0 (“rarely or none of the time [less than 1 day]” to 3 (“most or all of the times [5–7 days]”). A sample item is “I felt that I could not shake off the blues even with the help from my family”. The overall scores are summed to identify the severity and presented symptoms of depression. A composite score of the scale was used, with a higher score indicating a higher degree of depression. Based on a systematic review on CES-D, the cut-off score for subthreshold depression should be set as 20 [27]. The reliability of this scale in this study was high (α = 0.92).

### 2.2. Results

On average, participants spent 2 h and 22 min on SNS per day (*SD* = 1.74 h). A regression analysis was conducted with SNS usage as the predictor variable, age and gender as covariates, and depression symptoms as the outcome variable to derive a regression equation for calculating the amount of time of SNS usage when depression symptoms were at the threshold. Using the cut-off of 20 for the depression scale, when participants spent close to 3.5 h per day, they reached the cut-off for at risk of subthreshold depression. Similar method for calculating the cutoff time was applied for Studies 2 and 3.

No significant gender differences were found in time spent on SNS, *t*(345) = 0.64, *p* = 0.52; SE, *t*(345) = −0.78, *p* = 0.43; and depression, *t*(345) = 0.69, *p* = 0.49. Age was not correlated with any of the variables of interest, *ps* > 0.19.

**Figure 1:**
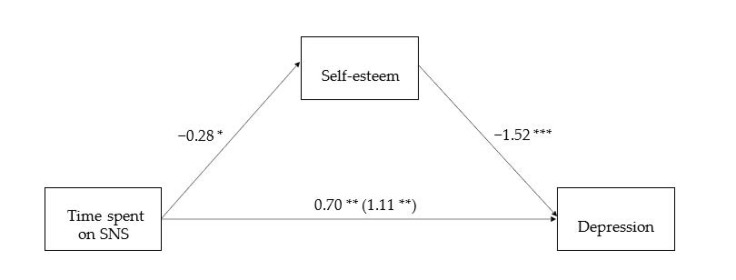
Self-esteem as mediator in the relation between Time spent on SNS and Depression. Note: *p* < 0.001 ***, *p* < 0.01 **, *p* < 0.05 *.

A simple mediation analysis using Model 4 of the PROCESS macro [28] with 5000 bootstrapped samples was conducted to test whether the relationship between time spent on SNS and depression was mediated by self-esteem, after controlling for gender and age (Figure 1). The PROCESS macro utilized bootstrapping, which is a resampling technique widely advocated for assessing mediation or indirect effects. This approach does not assume normality of the sampling distribution, and generates bias correction and acceleration confidence intervals, which can improve inferences of the mediation model [29].

The results showed that SNS predicted lower SE (*B* = −0.28, SE = 0.13, *t*(343) = −2.05, *p* = 0.04. Furthermore, SE was significantly associated with lower depression (*B* = −1.52, SE = 0.10, *t*(342) = −15.42, *p* < 0.001). The indirect effect of time spent on SNS to depression through SE was 0.42, 95% CI [0.02, 0.86]. Since the interval did not include zero, we concluded that the indirect effect was significant. This model accounted for 43.09% of variances of depression at *p* < 0.001.

### 2.3. Discussion

Study 1 supported hypothesis 1, in which SE mediated the relation between time spent on SNS and depression. Toma [30] argued that the self-affirmation theory [31] provides a theoretical framework on how SE is related to SNS usage: SNS allows users to build self-presentation by a favorable self-profile, and to build and maintain social connections with friends and family, which helps to satisfy their ego needs to craft their self-images and self-esteem. Therefore, it is worth studying the relation between SNS usage and SE. Extending on other past studies [32] which suggested that the more individuals tended to seek reassurance via Facebook, the lower their self-esteem were, which in turn, predicted higher thwarted belongingness and perceived burdensomeness, findings from the present mediation model supported that more time spent on SNS explained lower SE, which in turn, positively explained depression. 

Despite its cross-sectional nature and no causality can be drawn, future studies could test if including intervention elements on strengthening one’s SE, such as teaching Internet users to improve their SE by building healthy interpersonal relationships offline may help reduce depression symptoms.

Study 1 was not without limitations. For instance, usage of SNS was measured by a 30-min interval scale with 6.5 as the maximum. This might not capture the full range of usage, and Study 2 was designed to rectify this limitation.

## 3. Study 2

Building on the findings from Study 1 that self-esteem mediated time spent on SNS and depression, Study 2 investigated the mediating roles of FoMO, which tested our second hypothesis.

### 3.1. Methods

#### 3.1.1. Participants

A total of 180 Hong Kong Chinese undergraduate students aged between 18 to 26 (33.9% males, 66.1% females) with a mean age of 21.5 (*SD* = 1.67) years participated in this study. College students were chosen because they are frequent users of SNS.

#### 3.1.2. Procedures

Ethics approval was obtained from the Departmental Ethics Committee, of the university of the first author. Participants joined this study voluntarily without receiving incentives. Recent findings suggested that people who are high in FoMO are more likely to overuse smartphones to satisfy the need to stay continually connected and suffer from depressive symptoms [33], therefore, only participants who had smartphones would be included in subsequent analyses, and all of them fulfilled this requirement.

##### Basic Demographics

Basic demographics including age, gender (male = 0; female = 1), and whether they used smartphones were measured.

##### Time Spent on SNS

Participants were asked to put down the exact amount of time that they spent on SNS (including Facebook, Twitter, Instagram, etc.) per day.

##### Fear of Missing Out (FoMO)

Participants rated on a 10-item version of fear of missing out (FoMO) scale on a 5-point Likert scale [14]. It was used to determine the degree of fear of missing out, as reflected by the experienced fear, distress and anxiety in relation to being involved or uninvolved in rewarding events or activities of the participants. A composite score of the scale was used, with a higher score indicating higher degree of FoMO. A sample item is: “I get worried when I find out my friends are having fun without me”. The reliability of this scale in this study was high (α = 0.88).

##### Depression

Like in Study 1, depressive symptoms were assessed by the same 20-item CES-D that was described under Study 1 [26]. The reliability of this scale in this study was high (α = 0.91).

### 3.2. Results

Participants on average spent 4.03 h on SNS per day with a range from 30 min to 13 h (*SD* = 2.60 h). Using the same regression equation method as Study 1, when participants spent close to 2.5 h per day, they reached the cut-off for at risk of subthreshold depression.

No significant gender differences were found in time spent on SNS, *t*(178) = 1.37, *p* = 0.17; FoMO, *t*(178) = −1.02, *p* = 0.31. However, a significant difference was found for depression, *t*(178) = −2.14, *p* = 0.03 with males showing a higher level of depression symptoms. Age did not correlate with any of the variables of interest, *ps* > 0.18.

**Figure 2:**
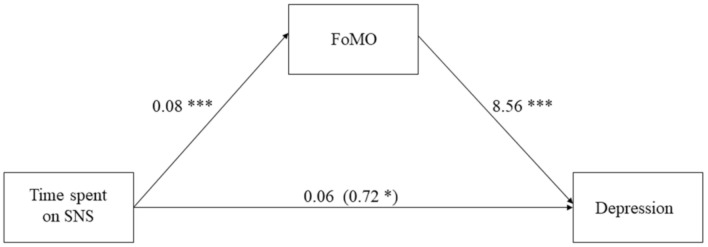
FOMO as mediator in the relation between Time spent on SNS and Depression. Note: *p* < 0.001 ***, *p* < 0.05 *.

We conducted a mediation analysis (model 4) to examine the effect of SNS usage on depression mediated by FoMO after controlling for gender and age [28]. The results of the analyses using PROCESS Model 4 showed that time spent on SNS was significantly related to higher FoMO (*B* = 0.08, SE = 0.02, *t*(176) = 3.57, *p* < 0.001), FoMO was positively associated with depression (*B* = 8.56, SE = 0.76, *t*(175) = 11.25, *p* < 0.001) (Figure 2).

The indirect effect of time spent on SNS to depression through FoMO was 0.67 with a bootstrap sample of 5000, SE = 0.18, 95% CI [0.33, 1.02], since the intervals did not include zero, we concluded that the indirect effect of FOMO was significant; this model accounted for R^2^ = 45.40% of variances of depression at *p* < 0.001.

### 3.3. Discussion

Study 2 supported hypothesis 2, FoMO mediated the relation between time spent on SNS and depression. Individuals who spent more time on SNS had a higher fear, distress and anxiety of “missing out” of not being included in rewarding experiences, which in turn, was positively related to their depression symptoms.

In Study 2, we found that FoMO mediated SNS usage and depression. The Self Determination Theory (SDT) proposes that individuals have to fulfill their basic needs of competence, autonomy and connectedness [14]. In study 2 of Przybylski and colleagues’ study [14], they found that people with a lower level of satisfaction of psychological needs tended to report higher levels of FoMO, and the authors concluded that FoMO arises from situational or chronic deficits in fulfilling these psychological needs.

People who are low in psychological need satisfaction in real life may turn to SNS to fulfil their needs. According to the fear-driven/compensatory-seeking hypothesis, people with social deficits and a higher social need have the expectation to compensate for these deficits and to reduce their fear of social isolation by using more SNS [34]. In other words, if people use SNS to reduce their social deficits and fear of isolation, this might result in the vicious cycle of having more FoMO, followed by the use of SNS. Then, FoMO was associated with more negative feelings, notably depression, as found in this study.

Again, because of the cross-sectional nature of the study, we cannot conclude causality from this study. It is suggested that more future studies can be conducted to find out the causal effect between SNS usage, FoMO and depression. For instance, future research can examine whether building a supportive social networking cycle beyond relying on SNS may reduce depression, and to investigate how different motives of SNS usage will impact well-being [35]. In addition, researchers could consider further investigating strategies, interventions, and educational programmes that reduce individuals’ FoMO, such as promoting “Joy of Missing Out” (JoMO) by disconnecting from SNS, and restricting access to smartphones [36], as to fill up the gap in the existing literature.

## 4. Study 3

Building on the findings from Studies 1 and 2, in Study 3, we investigated whether self-esteem and FoMO would mediate time spent on SNS and depression by simultaneously putting the two factors as mediators in the mediation model.

### 4.1. Methods

#### 4.1.1. Participants

233 Hong Kong Chinese undergraduate students aged between 18 to 33 (27.9% males, 72.1% females) with a mean age of 21.05 (*SD* = 1.80) years participated in this study. Three participants reported zero amount of SNS usage per day were excluded from the subsequent analysis.

#### 4.1.2. Procedures

Ethics approval was obtained (same as Study 1). Participants were recruited via mass emails to all students via intranet, with no limitations on years of study nor major being set. Upon consenting to the study, participants would complete an online questionnaire which includes the following measures. They received HK$50 (~USD7) for completing the questionnaire.

##### Basic Demographics

Basic demographics including age, gender (male = 0; female = 1), and whether they used smartphones were measured. 

##### Time Spent on SNS

Participants were asked to put down the exact amount of time that they spent on SNS (including Facebook, Twitter, Instagram, etc.) per day. 

##### Self-Esteem

Same as Study 1, participants completed the 10-item Rosenberg SE Scale on a 4-point Likert scale. Cronbach’s alpha was 0.87.

##### Fear of Missing Out (FoMO)

Same as Study 2, FOMO was measured by the same fear of missing out (FoMO) scale on a 5-point Likert scale utilized in Study 2 [14]. The Cronbach’s alpha of this scale was 0.88.

##### Depression

Depressive symptoms were assessed by the same 20-item CES-D that was described in the previous two studies [26]. The Cronbach’s alpha of this scale was 0.92.

### 4.2. Results

Participants on average spent 2.59 h on SNS per day with a range from 5 min to 9 h (*SD* = 1.63 h). Using the same regression equation method as Studies 1 and 2, when participants spent 2.54 h per day, they reached the cut-off for at risk of subthreshold depression.

No significant gender differences were found in time spent on SNS, *t*(228) = −0.80, *p* = 0.43; SE, *t*(228) = 1.04, *p* = 0.30; FoMO, *t*(228) = 1.58, *p* = 0.12. and depression, *t*(228) = −0.12, *p* = 0.91. Age did not associate with any of the variables of interest, *ps* > 0.23.

**Figure 3:**
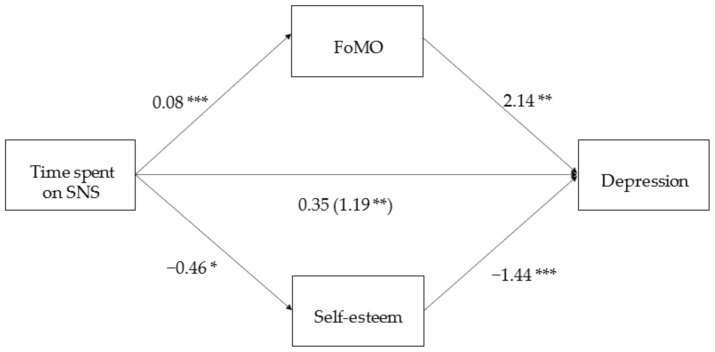
FoMO and Self-esteem as mediators in the relation between Time spent on SNS and Depression. Note: *p* < 0.001 ***, *p* < 0.01 **, *p* < 0.05 *.

We conducted a mediation analysis (model 4) to examine the effect of SNS usage on depression by simultaneously putting FoMO and SE as mediators and gender and age as covariates. The results of the analyses using PROCESS Model 4 showed that time spent on SNS was significantly related to higher FoMO (*B* = 0.08, SE = 0.03, *t*(225) = 2.67, *p* = 0.008), FoMO was positively associated with depression (*B* = 2.14, SE = 0.71, *t*(223) = 3.00, *p* = 0.003) (Figure 3).

The indirect effect of time spent on SNS to depression through FoMO was 0.17 with a bootstrap sample of 5000, SE = 0.10, 95% CI [0.02, 0.40], since the intervals did not include zero, we concluded that the indirect effect of FOMO was significant. Time spent on SNS was negatively associated with SE (*B* = −0.46, SE = 0.19, *t*(225) = −2.46, *p* = 0.01). Furthermore, SE (*B* = −1.44, SE = 0.12, *t*(223) = −12.33, *p* < 0.001) was negatively associated with depression. The indirect effect of time spent on SNS to depression through SE was 0.66 with a bootstrap sample of 5000, SE = 0.31 yielding a 95% CI of 0.02 to 1.21, as the interval did not include zero, we concluded that the indirect effect of self-esteem was significant. The whole model accounted for R^2^ = 51.70% of variances of depression at *p* < 0.001.

### 4.3. Discussion

Study 3 supported hypothesis 3, both SE and FoMO mediated the relation between time spent on SNS and depression. Individuals who spent more time on SNS were more prone to the feeling of “missing out” or not being included in rewarding experiences, which in turn, was positively related to their depression symptoms. At the same time, individuals who spent more time on SNS tended to have lower SE, while those who had lower SE tended to have more depression symptoms. Put together, both FoMO and SE are important mediators in explaining the relation between SNS usage and depression symptoms.

## 5. General Discussion

In general, our three studies supported that time spent on SNS was positively related to depression among Chinese college students. This paper contributed to the field by first supporting that SE mediated the relationship between SNS usage and depression in Study 1, FoMO mediated the relationship between SNS usage and depression in Study 2. When testing the competing mediators, FoMO and SE, to see how they contributed to the SNS usage and depression link in Study 3. Results showed that both FoMO and SE mediated the link between time spent on SNS and depression. Finally, we found that when participants spent about 3.5 h (Study 1), 2.5 h (Study 2), and 2.54 h (Study 3) on SNS, they reached the cutoff for at risk of subthreshold depression. However, by no means, for all participants who used SNS more than the aforementioned number of hours would reach subthreshold depression. The opposite was true that participants might still be depressed if they spent less than the cutoff hours. These findings contributed to the gap in the literature on SNS usage and depression in a Chinese context as well.

Our research has a few limitations. First, it is cross-sectional, so causal relations cannot be established. In addition, because of the cross-sectional nature of the data, it is unclear whether a change or reduction of SNS use will lead to a change in level of depression symptoms. Second, the gender ratio was imbalanced, there were more females than males in the three studies, with a female to male student ratio about 2.06:1. However, as participants were college students from Hong Kong, China, the gender ratio in the three studies were consistent with the imbalanced gender ratio in universities in Hong Kong, China, because in general, there are more female than male students (female to male student ratio ranging from 1.13:1 to 3.17:1) in universities in Hong Kong, China [37].

Third, our sample size was adequate at best. We set the target of the sample size for the three studies on the basis of power analyses (α = 0.05, 1 − β = 0.80) using an online tool developed by Schoemann and colleagues [38]. Overall, Studies 1 and 2 reached our targeted sample size, but sample size of Study 3 was slightly below what we targeted due to lower than expected participation rate in the data collection period. Although we still found significant results for all three studies, we shall interpret the results with caution, and we should also try to increase the sample size in future studies to maximize the power.

Despite its limitations, this paper contributes to the field by showing that SNS usage was positively related to depression in a Chinese population with a total sample of 760 participants. Cultural context may play an important role in social media involvement and interpretation of social connection, as Chinese people tend to place great emphasis on social connection and community value. The mediating roles of FoMO, and SE may be more evident as gathering information of what others are doing, and how others value oneself could be very valuable in Chinese culture. It is suggested that future studies should involve samples from different cultures, to allow cross-cultural comparisons to be made.

In addition, our findings supported the mediating role of SE between SNS usage and depressive symptoms. However, we only measured global SE in the current study, while some past studies suggested the importance of measuring other types of self-esteem, such as contingent self-esteem or contingent self-worth, which refers to self-esteem contingent on other’s approval. For instance, contingent self-worth was found to be positively related to time spent on Facebook, and negatively related to global SE [39]. Therefore, future studies can extend our understanding on SE by including other types of self-esteem in further understanding the mechanism between usage of SNS and depressive symptoms.

Despite its cross-sectional nature and no causality can be drawn, findings demonstrate that the link between SNS and depression is largely explained by the link between SNS usage, SE, and FoMO, which is in turn associated with depression. As psychopathologies (which includes depression) are linked to suicidal thoughts and behaviors, and psychological strains have been found to be related to depression, anxiety, and stress among Chinese students [40], it is important to have preventive measures to reduce depression. Results of this study provide initial practical implications for identifying individuals who are prone to depressive symptoms, by measuring their time spent on SNS, FoMO and SE. Future research directions should consider testing if intervention to reduce individuals’ FoMO and increase their self-esteem, and to spend appropriate time on social media use may help prevent youth suffering from more severe mental health issues.

## Data Availability

Data will be available upon request.
